# Real-life experience with remdesivir for treatment of hospitalized coronavirus disease 2019 patients: matched case-control study from a large tertiary hospital registry

**DOI:** 10.3325/cmj.2022.63.536

**Published:** 2022-12

**Authors:** Marko Lucijanić, Nikolina Bušić, Petra Bistrović, Ivan Papić, Marina Zelenika Margeta, Paško Babić, Mihaela Barčan, Antica Pasarić, Mirna Mustapić, Nevenka Piskač Živković, Maja Ortner Hadžiabdić, Tomo Lucijanić, Ivica Lukšić, Bruno Baršić

**Affiliations:** 1 Hematology Department, Dubrava University Hospital, Zagreb, Croatia; 2 Primary Respiratory and Intensive Care Center, Dubrava University Hospital, Zagreb, Croatia; 3 University of Zagreb, School of Medicine, Zagreb, Croatia; 4 Cardiology Department, Dubrava University Hospital, Zagreb, Croatia; 5 Pharmacy Department, Dubrava University Hospital, Zagreb, Croatia; 6 Pulmonology Department, Dubrava University Hospital, Zagreb, Croatia; 7 Department of Emergency and Intensive Care Medicine, Dubrava University Hospital, Zagreb, Croatia; 8 Department of Clinical Immunology, Allergology, and Rheumatology, Dubrava University Hospital, Zagreb, Croatia; 9 Endocrinology Department, Dubrava University Hospital, Zagreb, Croatia; 10 Centre for Applied Pharmacy, Faculty of Pharmacy and Biochemistry, University of Zagreb, Zagreb, Croatia; 11 Department of Maxillofacial Surgery, Dubrava University Hospital, Zagreb, Croatia

## Abstract

**Aim:**

To evaluate the association of remdesivir use and the survival of hospitalized patients with coronavirus disease 2019 (COVID-19).

**Methods:**

We retrospectively reviewed the medical records of 5959 COVID-19 patients admitted to our tertiary-level hospital from March 2020 to June 2021. A total of 876 remdesivir-treated patients were matched with 876 control patients in terms of age, sex, Charlson comorbidity index (CCI), WHO-defined COVID-19 severity on admission, and oxygen requirement at the time of remdesivir use.

**Results:**

Among 1752 COVID-19 patients (median age 66 years, 61.8% men), 1405 (80.2%) had severe and 311 (17.8%) had critically severe COVID-19 on admission. Remdesivir was given at a median of one day after hospital admission and at a median of eight days from the onset of symptoms. Overall, 645 (73.6%) patients received remdesivir before high-flow oxygen therapy (HFOT) or mechanical ventilation (MV), 198 (22.6%) after HFOT institution, and 83 (9.5%) after MV institution. Remdesivir use was associated with improved survival in the entire cohort (hazard ratio 0.79, *P* = 0.006). Survival benefit was evident among patients receiving remdesivir during low-flow oxygen requirement (hazard ratio 0.61, *P* < 0.001) but not among patients who received it after starting HFOT (*P* = 0.499) or MV (*P* = 0.380).

**Conclusion:**

Remdesivir, if given during low-flow oxygen therapy, might be associated with survival benefit in hospitalized COVID-19 patients.

Severe acute respiratory syndrome coronavirus 2 (SARS-CoV-2) infection affects multiple organ systems and, in a substantial proportion of patients, presents itself through severe or critical symptoms (1,2). Patients developing respiratory insufficiency require hospital admission and often critical or intensive care. Vaccination reduced the number of patients with severe disease presentation who require hospital admission, thus leading to better outcomes in those who developed severe disease despite having received the vaccine (3). However, vaccine hesitancy (4) and waning effects of vaccination (5) remain important problems for long-term coronavirus disease 2019 (COVID-19) control.

Remdesivir is the first antiviral drug to be approved for the treatment of severe or critical COVID-19 based on shortened time to recovery (6), but it demonstrated no clear survival benefit in the randomized controlled setting (7). Reports based on real-life cohorts of COVID-19 patients suggest survival benefit of remdesivir in patients with less severe disease (8-11). However, the results are inconsistent (12,13), and the reports are conflicting regarding certain aspects of remdesivir use, such as the length of hospital stay (13). Due to uncertainties regarding the efficacy of remdesivir among real-life patients burdened with comorbidities, we evaluated the association of remdesivir use with survival in a large real-life cohort of COVID-19 patients treated in our tertiary level institution.

## Patients and methods

We retrospectively reviewed the records of 5959 consecutive COVID-19 patients admitted to Dubrava University Hospital, Zagreb, from March 2020 to June 2021. Our institution was repurposed to treat exclusively the most severe cases of COVID-19 and patients with other medical emergencies concomitantly positive for SARS-CoV-2. All patients had positive polymerase chain reaction or antigen test with compatible clinical presentation at hospital admission. They were treated according to the contemporary guidelines, with the majority of them receiving low-molecular-weight heparin thromboprophylaxis and corticosteroids with various dose intensity at the treating physician’s discretion. Remdesivir was given to patients who developed respiratory insufficiency. In the majority of patients, it was administered intravenously at a standard dose (200 mg intravenously on day one, followed by 100 mg intravenously daily for five days). All patients were white adults. Clinical and laboratory parameters were evaluated at admission, whereas outcomes were assessed during and after the hospital stay as a part of a hospital registry project. The study was approved by the Institutional Review Board of Dubrava University Hospital (2021/2503-04).

Among 5959 treated patients, we identified 876 patients who received remdesivir and compared them with 876 matched controls who did not receive the drug. Matching was performed using the MedCalc statistical program tool for case-control matching and was based on age, sex, Charlson comorbidity index (CCI), and WHO-defined COVID-19 severity on admission. To account for the fact that remdesivir was given to patients with respiratory deterioration, matching was performed in a stepwise manner in several groups of patients based on the intensity of respiratory support at the time of remdesivir administration. In the first step, patients who received remdesivir during mechanical ventilation (MV) were 1:1 matched to patients who did not receive remdesivir but required MV. In the second step, patients who received remdesivir during high-flow oxygen therapy (HFOT) were 1:1 matched to patients not receiving the drug who required HFOT, followed by patients requiring low-flow oxygen therapy of various intensity in subsequent steps. The matching procedure was repeated several times for unmatched patients, with less stringent criteria on age and CCI to avoid a loss of patients treated with remdesivir for final analyses. These matching variables (older age, male sex, higher comorbidity burden, more severe COVID-19 presentation) were chosen because they are recognized negative prognostic parameters in patients with COVID-19. The level of oxygen support at the time of remdesivir use/maximum level of oxygen support during hospital stay in non-remdesivir-treated patients was selected as a matching parameter to alleviate biases arising from clinical judgments for remdesivir use. The outcomes of interest were length of hospital stay and survival up to 114 days from hospital admission.

### Statistical methods

Numerical variables were tested for normality of distribution with the Kolmogorov-Smirnov test. Numerical variables are presented as median and interquartile range (IQR) and compared with the Mann-Whitney U test. Categorical variables are presented as frequencies and percentages and compared with the Χ^2^ test. Survival analyses were based on the Kaplan-Meier method, and survival was compared with the Cox-Mantel version of the log-rank test (14). In-hospital mortality was evaluated from the time of hospital admission to death of any cause occurring during or after hospital stay in the period of 114 days (the length of hospital stay of the patient with the longest hospital stay). For particular analyses aimed to evaluate the moderation of survival association of remdesivir with drug-related parameters (level of oxygen requirement at the time of remdesivir administration and time from onset of symptoms to remdesivir administration), control patients who did not receive the drug were assigned the characteristics of their remdesivir counterparts. Multivariate survival analysis was performed with the Cox regression. *P* values <0.05 were considered significant. The analyses were performed with the MedCalc statistical software, version 20.110 (MedCalc Software Ltd, Ostend, Belgium).

## Results

### Patients’ characteristics

The study enrolled 1752 COVID-19 patients (1082 or 61.8% men): 876 treated with remdesivir and 876 matched control patients. The median age was 66 years, IQR 56-74. The median CCI was 3 points, IQR 2-4. A total of 16 (0.9%) patients had mild, 20 (1.1%) had moderate, 1405 had severe (80.2%), and 311 (17.8%) had critical COVID-19 on admission. During hospital stay, 646 (36.9%) patients required HFOT and 493 (28.1%) required MV. A total of 552 (31.5%) patients died. The median duration of hospital stay was 11 days, IQR 8-18.

Patients who were treated with remdesivir received the drug at a median of one day after hospital admission, IQR 1-2, and at a median of eight days from the onset of COVID-19 symptoms, IQR 6-10. The drug was given for five days or shorter in 823 (93.9%) patients and for longer than five days in 53 (6.1%) patients. A total of 645 (73.6%) patients received the drug before HFOT or MV support, 198 (22.6%) after the institution of HFOT, and 83 (9.5%) after the institution of MV support.

### Comparison of remdesivir-treated and matched control patients

Patients' characteristics are presented in Table 1. The remdesivir and control group were balanced regarding age, sex, CCI, WHO COVID-19 severity, and Eastern Cooperative Oncology Group (ECOG) functional status, C-reactive protein (CRP), ferritin, and procalcitonin on admission, and MV and HFOT support during hospital stay, as per the matching procedure (*P* > 0.05 for all comparisons). Despite similar overall comorbidity burden, remdesivir-treated patients were significantly more likely to be obese (40.9% vs 35.7%, *P* = 0.027), were less likely to have chronic renal insufficiency (4.7% vs 7.1%, *P* = 0.033), and had lower white blood cell (WBC) count on admission (median 7.5 vs 8.2 x10^9^/L, *P* < 0.001).

**Table 1:** Baseline clinical characteristics of remdesivir-treated and matched control patients

	Patients		
	remdesivir-treated (N = 876)	matched control (N = 876)	P
**Age** (years), median (IQR)	65 (56-74)	66 (57-74)	0.109
**Sex**			
female	335 (38.2)	335 (38.2)	1.000
male	541 (61.8)	541 (61.8)	
**Charlson comorbidity index**, median (IQR)	3 (1-4)	3 (2-4)	0.114
**Modified Early Warning Score**	3 (2-4)	3 (1-4)	0.289
**COVID-19 severity**			
mild	8 (0.9)	8 (0.9)	0.829
moderate	10 (1.1)	10 (1.1)	
severe	695 (79.3)	710 (81.1)	
critical	163 (18.6)	148 (16.9)	
**Length of stay (days)**, median (IQR)	12 (8-18)	11 (7-18)	<0.001
**HFOT during hospital stay**	320 (36.5)	326 (37.2)	0.766
**MV during hospital stay**	249 (28.4)	244 (27.9)	0.791
**ICU admission**	294 (33.6)	310 (35.4)	0.421
**ECOG functional status**, median (IQR)	2 (1-3)	2 (1-3)	0.244
**Arterial hypertension**	536 (61.2)	538 (61.4)	0.921
**Diabetes mellitus**	264 (30.1)	233 (26.6)	0.101
**Hyperlipoproteinemia**	182 (20.8)	183 (20.9)	0.953
**Obesity**	358 (40.9)	313 (35.7)	0.027
**Coronary artery disease**	82 (9.4)	84 (9.6)	0.870
**Atrial fibrillation**	88 (10)	100 (11.4)	0.354
**Prior venous thromboembolism**	26 (3)	32 (3.75)	0.423
**Chronic kidney disease**	41 (4.7)	62 (7.1)	0.033
**Chronic liver disease**	13 (1.5)	22 (2.5)	0.124
**Chronic obstructive lung disease**	41 (4.7)	51 (5.8)	0.284
**Active malignancy**	61 (7)	49 (5.6)	0.237
**C-reactive protein** (mg/L), median (IQR)	100.5 (55.5-157.3)	94.2 (42.9-165.2)	0.165
**Ferritin** (μg/L), median (IQR)	955 (561-1635)	900 (461-1581)	0.083
**White blood cells** (×10^9^/L), median (IQR)	7.5 (5.4-10.3)	8.2 (5.8-11.5)	<0.001
**Absolute neutrophils** (×10^9^/L), median (IQR)	5.9 (4.2-8.7)	6.5 (4.3-9.6)	0.009
**Absolute lymphocytes** (×10^9^/L), median (IQR)	0.73 (0.53-1.0)	0.8 (0.55-1.2)	0.016
**Hemoglobin** (g/L), median (IQR)	132 (120-143)	132 (119-143)	0.403
**Platelets** (×10^9^/L), median (IQR)	219 (169-284)	225 (164-299)	0.164
**Procalcitonin** (ng/mL), median (IQR)	0.19 (0.09-0.59)	0.21 (0.01-0.73)	0.219

*Abbreviations: IQR – interquartile range; HFOT – high-flow oxygen therapy; MV – mechanical ventilation; ICU – intensive care unit; ECOG – Eastern Cooperative Oncology Group.

### Duration of hospital stay and associations with survival

**Figure 1:**
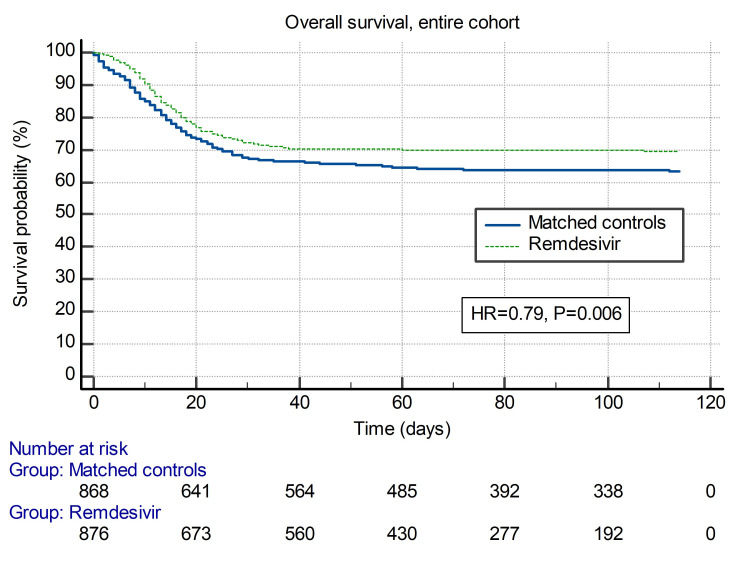
In-hospital survival in remdesivir vs matched control patients.

**Figure 2:**
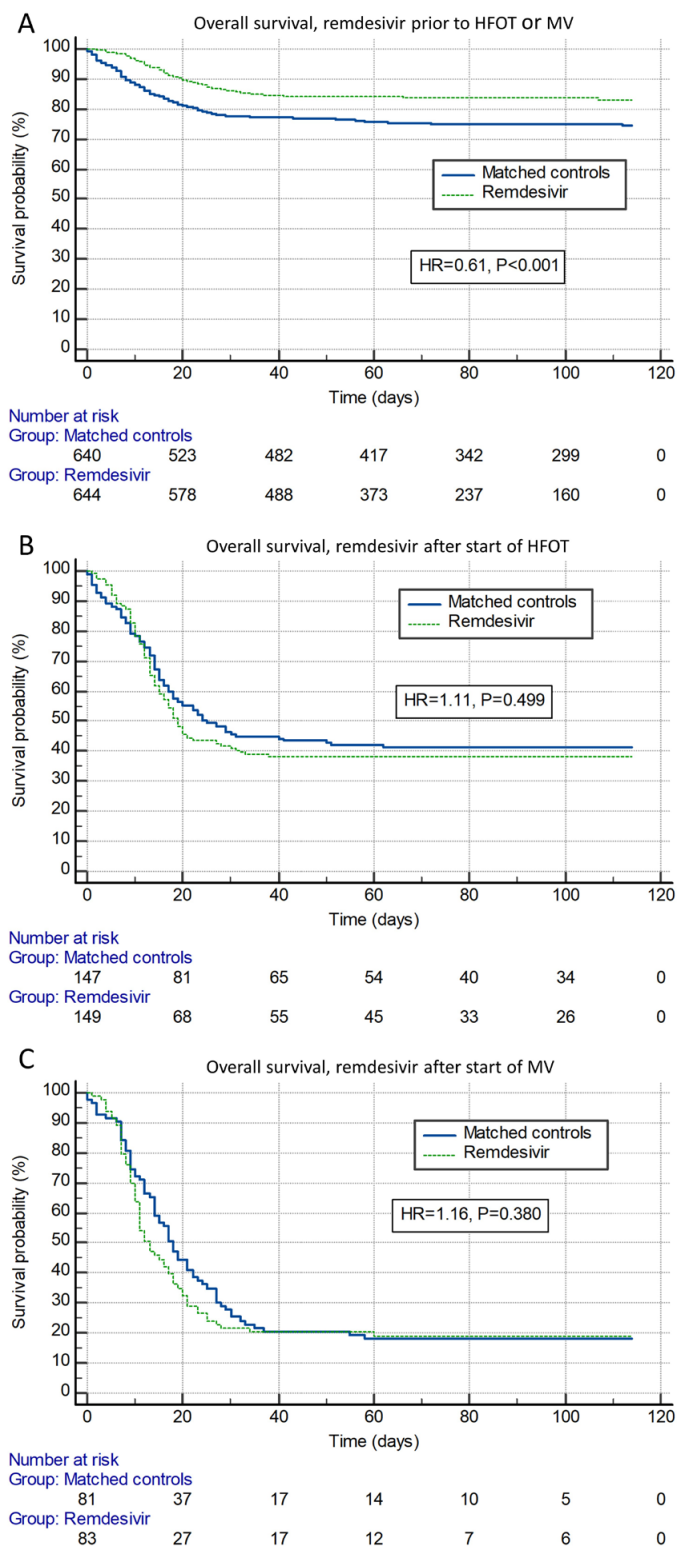
In-hospital survival in remdesivir vs matched control patients stratified according to the level of oxygen support at the time of remdesivir use. **A)** Remdesivir given before high-flow oxygen therapy (HFOT) or mechanical ventilation (MV), **B)** after start of HFOT, and **C)** after start of MV. HR – hazard ratio.

Patients who received remdesivir had longer hospital stay (median 12 vs 11 days, *P* < 0.001). The median follow-up in our cohort was 94 days. Remdesivir-treated patients had improved survival compared with the control group (70.8% vs 66.2%, HR 0.79, *P* = 0.006; Figure 1). Survival benefit was most pronounced among patients who received remdesivir before HFOT or MV (HR 0.61, *P* < 0.001, Figure 2A) but was not evident among patients who received the drug after the start of HFOT (HR 1.11, *P* = 0.499, Figure 2B) or MV (HR 1.16, *P* = 0.380, Figure 2C). The interaction between remdesivir use, level of oxygen requirement, and survival was significant (*P* < 0.001). Among patients receiving remdesivir before HFOT or MV, survival benefit was present in both those receiving the drug during the first seven days (HR 0.71, *P* = 0.048) and those receiving it eight or more days from the onset of COVID-19 symptoms (HR 0.54, *P* < 0.001) compared with control patients.

In the multivariate Cox regression analysis model (Table 2), remdesivir use remained protective of worse survival independently of older age, higher comorbidity burden, critical severity of COVID-19 on admission, higher WBC, higher CRP, and start of remdesivir treatment during lower oxygen requirement.

**Table 2:** Cox regression analysis model for in-hospital survival

	P	HR with 95% CI
**Remdesivir vs controls**	0.049	0.85 (0.72-0.99)
**Age** (years)	<0.001	1.03 (1.02-1.04)
**Male sex**	0.212	1.12 (0.94-1.33)
**Charlson comorbidity index**	<0.001	1.1 (1.06-1.15)
**Critical COVID-19 severity**	0.020	1.27 (1.04-1.54)
**ECOG functional status**	<0.001	1.33 (1.23-1.44)
**WBC** (×10^9^/L)	0.003	1.01 (1-1.02)
**CRP** (mg/L)	<0.001	1.002 (1.001-1.003)
**Remdesivir prior to HFOT or MV**	<0.001	0.27 (0.22-0.32)
**Remdesivir ≤7 days from symptoms onset**	0.144	0.88 (0.75-1.04)

*Abbreviations: HR – hazard ratio; CI – confidence interval; HFOT – high flow oxygen therapy; MV – mechanical ventilation; ECOG – Eastern cooperative oncology group; WBC – white blood cell count; CRP – C-reactive protein.

## Discussion

Our retrospective study based on a large real-life cohort of COVID-19 patients concomitantly treated with corticosteroids supports the previous findings that remdesivir, if given during low-flow oxygen support, might be associated with survival benefit in hospitalized COVID-19 patients. In addition, it suggests that the level of oxygen requirement, but not necessarily longer duration of symptoms, affects the drug efficacy. Remdesivir use might also prolong the hospital stay.

Insufficient therapeutic options at the beginning of the COVID pandemic made physicians resort to the use of treatment combinations such as azithromycin and hydroxychloroquine, which yielded very little benefit but had many side effects, some even detrimental (15). Several months into the pandemic, the first antiviral drug against the novel coronavirus – remdesivir – received conditional approval for use in COVID-19 patients. Now, after three years, even after the arrival of immunomodulatory drugs such as tocilizumab and baricitinib and conditionally approved nirmatrelvir/ritonavir combination, remdesivir remains the only fully approved antiviral treatment against SARS-CoV-2. Originally, it was intended for use in patients with severe or critical COVID-19 requiring oxygen supplementation up to the 15th day from the symptoms onset, which is how we prescribed it to the patients included in our study (16). Through further research and clinical practice, remdesivir showed best effects if administered as early in disease course as possible, resulting in a change in its indications. Nowadays, a five-to-ten-day remdesivir course is recommended to patients requiring oxygen and a three-day course, initiated up to seven days from symptoms onset, to those who do not require oxygen therapy but are at risk of severe COVID due to comorbidities (17). Remdesivir is also often used in immunocompromised patients to disrupt continuous SARS-CoV-2 replication due to the nature of their underlying disease (18).

A growing pool of retrospective studies reports survival benefit in patients who start the drug while having lower oxygen requirement. Retrospective analyses that compare remdesivir-exposed to non-exposed patients cannot be straightforwardly interpreted due to missing data, the inclusion of younger patients with lower comorbidity burden and without contraindications for remdesivir use, as well as due to confounding with measured and unmeasured variables. At the same time, remdesivir is commonly given to patients with respiratory deterioration, in whom it may be less efficient, and our results are in line with the majority of previously published real-life studies. Such observations raise concerns whether patients with more advanced oxygen requirement benefit from the drug. It is unknown, however, whether these patients would have fared even worse without remdesivir and to what extent remdesivir might modulate other aspects of inflammation besides viral load reduction. Remdesivir is a prodrug, and its metabolites are adenosine analogues with a longer half-life than adenosine (19), which might have a number of off-target effects. Adenosine may exert chronotropic effects on the heart (20) and modify immune response (21,22). Besides kidney and hepatic toxicities (23), remdesivir use in COVID-19 patients has been associated with development of bradycardia (24,25), repolarization abnormalities (26), and a higher frequency of bacteremia (27), all of which are phenomena with uncertain mechanisms and various prognostic impact on the affected patients. Thus, there are uncertainties regarding remdesivir use in COVID-19 patients with cardiovascular and other comorbidities, bacterial co-infections, etc. It was recently suggested that remdesivir use reduces mortality in patients with atrial fibrillation (28), an effect that is possibly associated with a better heart rate control (25). In the absence of robust data from randomized controlled trials, real-life exploratory studies are very important as they provide insights into factors that might moderate the efficacy and safety of remdesivir use, especially in patients with comorbidities, who are not typically included in randomized trials. Remdesivir use may also be associated with a longer hospital stay. This finding is contrary to initial results from randomized studies (7), but was previously reported (13). Factors introduced with remdesivir administration, such as complications of treatment and adherence to pre-planned drug schedule in otherwise stabilized patients might play a role. It should be noted, however, that median difference of only one day might not be clinically significant.

The main limitations of our work are single-center experience and retrospective study design. No association of remdesivir use with respiratory deterioration could be evaluated from our data set due to the matching approach that balanced patients with maximum oxygen requirement to avoid over-representation of patients requiring HFOT and MV in the remdesivir group. Due to retrospective design, residual confounding could not have been avoided despite efforts to balance the two groups regarding factors important for survival. The main strength of our study is a large real-life cohort of mostly severe or critical COVID-19 patients with chronic and acute comorbidities, representative of a high-volume tertiary referral center.

In conclusion, remdesivir use might be associated with improved survival in hospitalized COVID-19 patients, a phenomenon evident only among patients who started remdesivir while being treated with low-flow oxygen supplementation and not among those who started remdesivir after institution of HFOT or MV.
